# The value of nonoperative versus operative treatment of frail institutionalized elderly patients with a proximal femoral fracture in the shade of life (FRAIL-HIP); protocol for a multicenter observational cohort study

**DOI:** 10.1186/s12877-019-1324-7

**Published:** 2019-11-08

**Authors:** Pieter Joosse, Sverre A. I. Loggers, C. L. P. (Marc) Van de Ree, Romke Van Balen, Jeroen Steens, Rutger G. Zuurmond, Taco Gosens, Sven H. Van Helden, Suzanne Polinder, Hanna C. Willems, Esther M. M. Van Lieshout, on behalf of the FRAIL-HIP study group, Lisanne Balemans, Frank W. Bloemers, Janneke Bos, Jantien Brouwer, Bart J. Burger, Judella O. Daal, Annemarieke De Jonghe, Matthea Dijkshoorn, Michael J. R. Edwards, Ellen A. Elbrecht, Miriam C. Faes, Elvira R. Flikweert, Robert D. A. Gaasbeek, Olivia C. Geraghty, J. Carel Goslings, J. Han Hegeman, Mischa M. Hindriks, Michel Holla, André Janse, Joris A. Jansen, Simone J. M. Jong, Paul J. C. Kapitein, Y. V. ( I Jdo) Kleinlugtenbelt, Barbara E. Kreis, Rover Krips, Koen W. W. Lansink, Michiel Leijnen, Pieter H. W. Lubbert, M. Cor Luyten, Franceso U. S. Mattace Raso, Marieke C. Meinardi, Joris Mellema, Roland M. H. G. Mollen, Majon Muller, Joost C. Peerbooms, Kees-Jan Ponsen, Rudolf W. Poolman, Miruna Popescu, Albert F. Pull ter Gunne, Bas J. Punt, Gert R. Roukema, Hilde I. F. Royen, Jeanine Schukking, Josje Snoek, Charles T. Stevens, Dieneke van Asselt, Alexander H. Van der Veen, Detlef Van der Velde, Bart A. Van Dijkman, Paul J. Van Koperen, Job L. C. Van Susante, Romuald Van Velde, M. Remmelt Veen, Michael H. J. Verhofstad, Ralf W. Vingerhoets, Dagmar I. Vos, Hugo H. Wijnen, Judith Wilmer, Jasper Winkelhagen, Johan F. H. Wold, Robbert A. Zandbergen, G. ( Bert) Ziere

**Affiliations:** 1 Department of Surgery, Noordwest Ziekenhuisgroep, P.O Box 501, 1800 AM Alkmaar, The Netherlands; 2 Trauma Research Unit, Department of Surgery, Erasmus MC, University Medical Center Rotterdam, P.O. Box 2040, 3000 CA Rotterdam, The Netherlands; 3 Department Trauma TopCare, Elisabeth-TweeSteden Ziekenhuis, P.O. Box 90151, 5000 LC Tilburg, The Netherlands; 4 Department of Public Health and Primary Care, Leiden University Medical Center, Hippocratespad 21, P.O. Box 9600, 2300 RC Leiden, The Netherlands; 5 Department of Orthopaedic Surgery, Dijklander Ziekenhuis (location Westfriesgasthuis), P.O. Box 600, 1620 AR Hoorn, The Netherlands; 6 Department of Orthopaedic Surgery, Dijklanders Ziekenhuis (location Waterland Ziekenhuis), P.O. Box 250, 1440 AG Purmerend, The Netherlands; 7 Department of Orthopaedic Surgery, Isala, P.O. Box 10400, 8000 GK Zwolle, The Netherlands; 8 Department of Orthopaedic Surgery, Elisabeth-TweeSteden Ziekenhuis, P.O. Box 90151, 5000 LC Tilburg, The Netherlands; 9 Department of Surgery, Isala, P.O. Box 10400, 8000 GK Zwolle, The Netherlands; 10 Department of Public Health, Erasmus MC, University Medical Center Rotterdam, P.O. Box 2040, 3000 CA Rotterdam, The Netherlands; 11 Geriatrics Section, Department of Internal Medicine, Amsterdam UMC location AMC, P.O. Box 22660, 1100 DD Amsterdam, The Netherlands

**Keywords:** Elderly, Frailty, Hip fracture, Proximal femoral fracture, Nonoperative treatment, Nursing home, Operative treatment, Palliative care, Quality of life, Surgery

## Abstract

**Background:**

Proximal femoral fractures are strongly associated with morbidity and mortality in elderly patients. Mortality is highest among frail institutionalized elderly with both physical and cognitive comorbidities who consequently have a limited life expectancy. Evidence based guidelines on whether or not to operate on these patients in the case of a proximal femoral fracture are lacking. Practice variation occurs, and it remains unknown if nonoperative treatment would result in at least the same quality of life as operative treatment. This study aims to determine the effect of nonoperative management versus operative management of proximal femoral fractures in a selected group of frail institutionalized elderly on the quality of life, level of pain, rate of complications, time to death, satisfaction of the patient (or proxy) and the caregiver with the management strategy, and health care consumption.

**Methods:**

This is a multicenter, observational cohort study. Frail institutionalized elderly (70 years or older with a body mass index < 18.5, a Functional Ambulation Category of 2 or lower pre-trauma, or an American Society of Anesthesiologists score of 4 or 5), who sustained a proximal femoral fracture are eligible to participate. Patients with a pathological or periprosthetic fractures and known metastatic oncological disease will be excluded. Treatment decision will be reached following a structured shared decision process. The primary outcome is quality of life (Euro-QoL; EQ-5D-5 L). Secondary outcome measures are quality of life measured with the QUALIDEM, pain level (PACSLAC), pain medication use, treatment satisfaction of patient (or proxy) and caregivers, quality of dying (QODD), time to death, and direct medical costs. A cost-utility and cost-effectiveness analysis will be done, using the EQ-5D utility score and QUALIDEM score, respectively. Non-inferiority of nonoperative treatment is assumed with a limit of 0.15 on the EQ-5D score. Data will be acquired at 7, 14, and 30 days and at 3 and 6 months after trauma.

**Discussion:**

The results of this study will provide insight into the true value of nonoperative treatment of proximal femoral fractures in frail elderly with a limited life expectancy. The results may be used for updating (inter)national treatment guidelines.

**Trial registration:**

The study is registered at the Netherlands Trial Register (NTR7245; date 10-06-2018).

## Background

Proximal femoral fractures are amongst the most common fractures in the elderly [1] with the incidence rate increasing with age. In the Netherlands 20,000 patients are admitted with a proximal femoral fracture to the hospital each year [2, 3]. This number is expected to rise because of our ageing society. Approximately 20% live in a nursing home prior to the fracture. Proximal femoral fractures result in activities of daily living (ADL) dependence and increased morbidity, strongly diminishes the patients’ (health-related) Quality of Life (QoL) and total health care costs [4–7].

A proximal femoral fracture is also strongly associated with mortality. Over 30% of patients die within the first year and 8–13.3% die within the first month [8–10]. Mortality is highest among elderly with both physical and cognitive comorbidities [9, 10]. A 36 and 55% mortality rate within 6 months after fracture has been reported for institutionalized patients and patients with advanced dementia, respectively [11, 12]. This shows that the hip fracture is merely a symptom of the frail status of these patients and the start of cascade breakdown at the end of life.

Operative treatment allows early mobilization, is effective in pain relief, and is believed to prevent secondary complications like pneumonia, urinary tract infection, and pressure sores. However, surgery will not prevent frail patients from developing aforementioned complications. Instead, patients may develop additional surgical complications like bleeding, infection, and non-union, necessitating further (operative) interventions [13]. Hospital admission after a proximal femoral fracture itself can provoke cognitive impairment or delirium in these patients who often live in delicate equipoise in an institutionalized setting.

Although surgery is considered a good analgesic option, it is not the only analgesic option. Moreover, surgery also has disadvantages. It is questionable whether surgery is beneficial in terms of quality of life and whether surgery is satisfactory for family and caretakers involved. The World Health Organization (WHO) definition for palliative care supports a holistic approach in facing life-threatening illness, which is a proximal femoral fracture in a frail elderly patient. Randomized trials comparing operative and nonoperative management in institutionalized elderly are scarce, due to ethical issues, and focus on mortality instead of quality of life.

Guidelines have no strict advice for frail elderly with a proximal femoral fracture, as a result of lack of evidence on palliative care in these patients. The National Institute for Health and Care Excellence (NICE) advises to discuss if patients are open to hospital admission and possible surgery [14]. Dutch guidelines advocate operative treatment in patients with a life expectancy beyond 6 weeks [2, 15]. This leads to practice variation. Current practice is that over 90% of femoral fracture patients that presented to hospital are operated, even if they are institutionalized and have a limited life expectancy [16].

A systematic review of the literature on nonoperative management versus operative management of proximal femoral fractures in frail elderly resulted in 7 studies that met the eligibility criteria [17–24]; All were nonrandomized, five were retrospective studies. A total of 1189 patients were included, 242 (20.3%) were treated nonoperatively. The mean age ranged from 76.9–101.8 years. The data indicated an unadjusted pooled odds ratio of 3.95-fold and 3.84-fold higher 30-day and 1-year mortality for nonoperative versus operative management. Data also showed that mortality was affected by the number of comorbidities. None of the comparative studies examined (health-related) quality of life, degree of frailty, and costs. The literature review shows a lack of high quality evidence on the true effect of nonoperative versus operative management on quality of life, pain, mortality, complications, and costs in the first 6 months after trauma in frail institutionalized elderly. Those answers are needed to aid patients and health care providers in decision-making for surgical repair, particularly in frail elderly patients. The above shows that the best treatment is not known, and supports the need for high quality evidence. Evidence supporting (non)operative management in terms of quality of life, pain, complications, and mortality is lacking.

It is common practice to decide on treatment following a shared decision process, but still nonoperative treatment is uncommonly used. It is unclear to what extent patients or their relatives currently have a say in the treatment decision.

This study aims to investigate the effect of nonoperative management versus operative management of proximal femoral fractures in a selected group of frail institutionalized elderly with regards to quality of life and clinical outcomes. Treatment decision will be reached following a structured shared decision process, in which pros and cons of both operative and nonoperative management are discussed with patients, their relatives, and all relevant care providers involved.

## Methods/design

### Aim of the study

This study aims to investigate the effect of nonoperative management versus operative management of proximal femoral fractures in a selected group of frail institutionalized elderly on the quality of life, level of pain, rate of complications, time to death, satisfaction of the patient (or proxy) and the caregiver with the management strategy, and health care consumption.

### Study design and setting

This study will be a two-arm non-randomized (observational) multicenter cohort study and an economic evaluation alongside. The study will be conducted with a non-inferior design. Relevant societies for surgery (Dutch Association of Surgery (NVvH), Dutch Association of Trauma Surgery (NVT), Dutch Orthopaedic Association (NOV), and Dutch Association of Orthopaedic Surgery (NVOT)), clinical geriatrics (Dutch Association of Clinical Geriatrics (NVKG)), elderly care (Association of Elderly Care Physicians (VERENSO)), the Dutch Patient Federation, and two committee members of the Dutch guideline for treatment of proximal femoral fractures are represented in the study team. Patients will be recruited from several hospitals throughout The Netherlands. The following 26 hospitals will participate: Albert Schweitzer Ziekenhuis, Alrijne Ziekenhuis, Amphia Ziekenhuis, Amsterdam UMC (location VUmc), Bernhoven, Catharina Ziekenhuis, Deventer Ziekenhuis, Dijklander Ziekenhuis (location Westfriesgasthuis), Dijklander Ziekenhuis (location Waterland Ziekenhuis), Elisabeth TweeSteden Ziekenhuis, Erasmus MC, Flevo Ziekenhuis, Isala, Maasstad Ziekenhuis, Meander MC, Noordwest Ziekenhuisgroep, OLVG, Radboud UMC, Rijnstate, Rode Kruis Ziekenhuis, St. Antonius Ziekenhuis, Streekziekenhuis Koningin Beatrix, Tergooi, Ziekenhuis Gelderse Vallei, Ziekenhuis Tjongerschans, and Zorggroep Twente.

The study has started in September 2018. The recruitment period will be 24 months, with a follow-up of 6 months. The results of the study are expected mid-2021.

### Study registration

The study is registered at the Netherlands Trial Register (NTR7245; date 10-06-2018).

### Study population and eligibility criteria

This study focuses on frail institutionalized elderly who have a limited life expectancy and fracture their proximal femur. In order to be eligible to participate in this study, a subject must meet all of the following criteria:

1. Frail institutionalized elderly person (i.e., 70 years or older, living in a nursing home pretrauma, who either:
  - is malnutritioned (i.e., cachexia or a Body Mass Index, BMI, of < 18.5 kg/m^2^); or
  - has mobility issues with increased risk of falling pretrauma (i.e., Functional Ambulation Category (FAC) 2 or less) *; or
  - has severe comorbidities (American Society of Anesthesiologists (ASA) class 4 or 5).
2. Acute proximal femoral fracture, confirmed on X-ray or CT-scan
3. Provision of informed consent by patient or proxy

* The FAC is a functional walking test that evaluates ambulation ability [25, 26]. This 6-point scale assesses ambulation status by determining how much human support the patient requires when walking, regardless of whether or not they use a personal assistive device. FAC category 2 are patients who require manual contact of no more than one person during ambulation on level surfaces to prevent falling, consisting of continuous or intermittent light touch to assist balance or coordination. Patients categorized as 0 (nonfunctional ambulatory), 1 (dependent for continuous and necessary physical assistance) to 2 due to a temporary and easily resolvable problem will not be eligible.

A potential subject who meets any of the following criteria will be excluded from participation in this study:

1. Bilateral proximal femoral fractures
2. Periprosthetic fracture
3. Fracture diagnosed more than 7 days after trauma
4. Patients with a known metastatic disease and a confirmed pathological fracture of the proximal femur
5. Insufficient comprehension of Dutch language to understand rehabilitation programs and other treatment information (this applies to the person signing consent, being either the patient or proxy)
6. Participation in another surgical intervention or drug study that might influence any of the outcome parameters

### Treatment of participants

#### Shared decision process

The decision for type of treatment (operative or nonoperative) is achieved by shared decision making (SDM). Shared decision making is based on determining the goals of care for the patient [27]. The purpose of goals of care is to safeguard patients who are unlikely to benefit from operative treatment, especially in patients in the palliative or terminal phase. Goals of care discussion with these frail elderly patients, or in case of cognitive impairment, with a proxy at the onset of proximal femoral fracture are useful in shared decision making. Although some form of SDM is also used in current practice, this study will use a much more structured approach towards this process. Prior to start of treatment, patients will be informed in an objective and structured way about what either treatment arm of the study involves and what complications may occur in either treatment arm. Both for nonoperative and operative management, a full forecast is given verbally and on paper. Combined with the preferences and objections of the patients or their proxy, this is the starting point of a shared decision process. All principal health care providers (i.e., a trauma or orthopedic surgeon, elderly care physician, and geriatrician if possible) as well as the patient and his/her relative(s) or legal representative will take part in this shared decision process. The patients perspective will play a key role in the decision process.

#### Nonoperative management group

The intervention group will be subjected to nonoperative management of the proximal femoral fracture. This group is referred to as nonoperative group. The aim is to transfer patients back to their nursing home location as soon as reasonably possible after treatment decision has been made. Treatment is focused on patient comfort and consists of pain control (often using opioids), with breathing support and physical therapy and occupational therapy consultation (for optimal lying and sitting position) as deemed necessary by the multidisciplinary treatment team. Anti-decubitus mattresses beds and other measures to prevent or treat complications will be used as needed. Progression of comorbidities will also be monitored closely. The general strategy and outline for treatment will be the same for all patients. However, as treatment needs to be tailored to the individual patient’s needs, standardization of treatment is not possible. Relevant details of treatment will be registered for possible use in the statistical analysis.

#### Operative management group

Patient opting for operative treatment, will have operative management of the proximal femoral fracture (if no medical contra-indications or objections by treating physician). The choice of implant will depend on the fracture type, and will adhere to the Dutch guideline for hip fracture treatment [2]. In general, intracapsular femoral neck fractures will be fixed with a (hemi)arthroplasty or screw fixation, and extracapsular pertrochanteric fractures with a dynamic hip screw, dynamic compression screw, or an intra-medullary fixation. Perioperative care will be according to local hospital protocols in order to have a real-life situation. As soon as reasonably possible after surgery, patients will return to their nursing home for further (palliative) care. The principles for palliative care are the same as described for the nonoperative group. Relevant details of treatment will be registered for possible use in the statistical analysis.

### Outcome measures and data collection

The primary outcome measure is the quality of life as reported by the patient or proxy. The EuroQoL-5D (EQ-5D) is a standardized instrument for use as a measure of health outcome. It is among the most commonly used instruments for femoral fracture patients [28, 29]. EQ-5D use is recommended for the assessment of quality of life in trauma patients, especially for economic assessments [30, 31]. The EQ-5D-5 L descriptive system consists of five dimensions of health (mobility, self-care, usual activities, pain/discomfort anxiety/depression), each with five possible answers. It is available in a self-report and a proxy version. The patients’ EQ-5D-5 L health status will be converted into utility scores using the Dutch tariff [32]. Utility scores rang from zero to one, with lower scores indicating poorer quality of life.

The following secondary outcome measures will be used:

1. **Quality of life, measured with the QUALIDEM;** QUALIDEM (DL010) measures quality of life in persons with dementia (regardless of severity) who live in a nursing home or elderly care facility [33]. The instrument has been developed by the Trimbos Institute and VUmc/EMGO-institute, department of Psychiatry and department of Social and Elderly Care [34]. The QUALIDEM consists of 37 items about the last 7 days rated on a four-point Likert scale. Items are divided into nine subscales (i.e., care relationship, positive affect, negative affect, restless tense behavior, positive self-image, social relations, social isolation, feeling at home, and having something to do). Caregivers complete the questionnaire, which has acceptable psychometric properties [33, 35].
2. **Level of pain and analgesic drug use**: The level of pain will be determined using the Pain Assessment Checklist for Seniors with Limited Ability to Communicate (PACSLAC), which was designed to be a clinically useful scale for assessing pain in patients with dementia [36]. This instrument has been shown to be among the instruments with the strongest psychometric properties for measuring pain and lack of comfort in elderly in a long-term care setting [37]. The instrument is a 24-item questionnaire covering three sub-scales (facial and vocal expression, resistance/defense and social-emotional aspects/mood of the patient). A validated Dutch version (PACSLAC-D) is available [38, 39]. Trained caregivers can use the instrument for scoring the presence of pain. The total score is a summation of the 24 items that are each scored as present (1 point) or absent (0 points). The total score thus ranges from 0 to 24. A score of 4 points or higher indicates pain. In addition to the level of pain we will also register the amount of analgesic medications used. All analgesic medication given will be registered from the patients’ medical files. Opioids will be combined. Opioid use will be defined as daily oral morphine need. Daily narcotic use will be calculated using the equivalence scale for 30 mg/day oral morphine. Calculation of the equivalent morphine dosage allows for comparison between the two treatment groups.
3. **Time to death**: Mortality including (presumed) cause of death will be registered. This will be expressed as mean (or median, as applicable) time to death as well as the rate of mortality within 1 week, 30 days, 3 months, and 6 months.
4. **(Surgical) complications**: This will include general complications that may occur in both groups (e.g*.*, urinary tract infection, pneumonia, pressure sores, delirium, and comorbidity related complications like cardiac arrhythmia, aspiration during intubation, CVA) as well as early post-operative complications (e.g., hematoma, bleeding, blood loss, wound leakage, superficial infection, epidural bleeding due to spinal anesthesia) and late post-operative complications (e.g., deep infection, dislocation, or implant failure, blood loss requiring transfusion < 48 h after surgery). Complications will be categorized for level of severity and treatment necessity according to the Clavien-Dindo classification [40].
5. **Satisfaction of the patient (or proxy) with the chosen treatment**: At the last study visit, the patient (or proxy, as applicable) as well as the patient’s caregiver will be asked to complete an 11-point Numeric Rating Score asking about their satisfaction with the chosen treatment. Herein, 0 = extremely dissatisfied and 10 = extremely satisfied. For patients who have died, their relatives and caregiver will be asked to complete this Numeric Rating Scale. Patients relatives will also be asked to participate in an interview about the **Quality of Dying and Death (QODD)**. This is a 17-item interview-based questionnaire asking about 17 end-of-life priorities. Each item includes a filter question reporting what actually occurred during the final period of the decedent’s life (and how often), followed by a rating of what occurred (using a scale ranging from 0 (‘terrible experience’) to 10 (‘almost perfect experience’) [41, 42].
6. **Satisfaction of the patient’s caregiver with the chosen treatment** (11-point Numeric Rating Score, ranging from zero (extremely dissatisfied) to 10 (extremely satisfied).
7. **Health care resource utilization (with associated costs)**; relevant data will be collected using a questionnaire that is based on the validated Medical Consumption Questionnaire (iMCQ; institute of Medical Technology Assessments iMTA, Erasmus University, Rotterdam. NL) iMCQ details medical specialist care, physical therapy, hospitalization, nursing home, medication use, and other costs directly associated with diagnosis, treatment, and rehabilitation. Health care use and associated costs until 6 months after trauma will be measured in accordance with economic guidelines [43]. Cost prices of the standardized referral strategy will be determined by bottom-up micro-costing method. The incremental cost-utility ratio of nonoperative management versus operative management will be expressed as costs per Quality Adjusted Life Years (QALY) (EQ-5D utility score) gained, with confidence ellipses and acceptability curves. A cost-effectiveness analysis, with QUALIDEM score as outcome measure, will also be done.

### Other study parameters

In order to assess whether the treatment groups are similar with respect to baseline characteristics, several patient characteristics, injury characteristics, details of the shared decision process, and treatment characteristics will be collected.

#### Patient characteristics

- Age
- Gender
- BMI
- ASA grade
- Tobacco consumption at baseline
- Charlson Comorbidity Index (CCI) [44]
- Medication use prior to trauma
- FAC score
- Katz Index of Independence in Activities of Daily Living (KATZ-ADL score)

#### Injury characteristics

- Affected side
- Type of fracture
- Additional injuries

#### Details of shared decision process

- Participants in process
- Treatment goals of each participating party
- Relevant details of shared decision process

#### Treatment characteristics

- Hospital admission
- Surgical delay
- Name of surgeon
- Primary and secondary surgeon (resident or staff surgeon)
- Duration of surgery
- Type of anesthesia
- Peripheral nerve block
- Details of nonoperative treatment
- Type of implant used
- Peroperative complications
- Mobilization orders
- Anti-decubitus mattress

### Recruitment and informed consent

Eligible persons presented to the Emergency Department (ED) with a proven proximal femoral fracture will be informed about the study in the ED or upon admission at the surgical or geriatric ward. After an explanation of the study, they will receive written information and a consent form from the attending physician, the clinical investigator, or a research assistant. Patients meeting all inclusion criteria and none of the exclusion criteria will be recruited in the hospital within 48 h after hospital admission. Should patients not be able to sign informed consent themselves, a legal representative (proxy) will receive verbal and written information about the study by the attending physician, the clinical investigator, or a research assistant, and will be asked to consent with participation of the patient. After signing informed consent by patient or proxy, the shared decision process will be carried out using a structured approach. This will result in identifying the primary treatment that is deemed best for the individual patient. Participation in this study is on a voluntary basis. If patients (or proxy on their behalf) do not wish to participate, they can do so without specifying why. Deciding not to participate in the study will not affect regular treatment and follow-up care. Participants are allowed to withdraw from the study at any time after they have given their written consent.

### Study procedures and follow-up

**Fig. 1:**
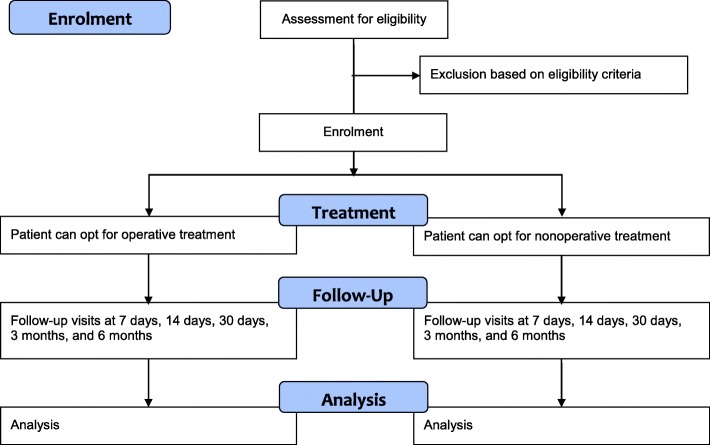
Flow of participants through the study

Patients will be followed until 6 months after trauma or until death, whichever comes first. In addition, at the 6-month follow-up contact, the researcher or research assistant will document any secondary intervention that may be planned for the patient. A schedule of events is shown in Table 1. A flow chart is shown in Fig. 1.

**Table 1:** Schedule of events and follow-up

Radiographs & Event forms	Screening	Enrolment	7 days (4–10 days)	14 days (11–17 days)	30 days (23–37 days)	3 months (11–15 weeks)	6 months (6–7 months)
X-ray or CT-scan	X						
Screening	X						
Informed Consent		X					
Shared Decision		X					
Baseline Data			X				
General Clinical FU			X	X	X	X	X
QoL (EQ-5D and QUALIDEM)			X^a^	X	X	X	X
PACSLAC and Analgesic use			X^a^	X	X	X	X
Complications and mortality			X	X	X	X	X
(Secondary) Interventions			X	X	X	X	X
Satisfaction of patient (or proxy)			X^b^	X^b^	X^b^	X^b^	X
QODD			X^b^	X^b^	X^b^	X^b^	X^b^
Satisfaction of caregiver			X^b^	X^b^	X^b^	X^b^	X
Health Care Use			X^a^	X	X	X	X
Early Withdrawal			^c^	^c^	^c^	^c^	^c^

EQ-5D is completed by proxy and (if possible) by patients themselves

^a^Asking for current and pre-trauma status; ^b^Only if patients died prior to that follow-up moment; ^c^Only if applicable

Following enrolment, treatment (either nonoperative or operative management) will be decided using a shared decision approach as mentioned above. Data will be acquired at 7, 14, and 30 days, and at 3 and 6 months after trauma.

Baseline data will be collected from the patient’s medical files or by interviewing the patient as soon as possible, but no later than at the visit after enrolment. At each follow-up visit, the coordinating researcher or research assistant will visit the patient. He will ascertain patient status (i.e., treatment details including analgesics and other health care use, mobility of the patients, adverse events/complications, secondary interventions, and will verify information within medical records). At each visit patients (or proxy) will be asked to complete a questionnaire for the EQ-5D. At those visits, a caregiver will be asked to complete the QUALIDEM and PACSLAC-D.

At the final visit at 6 months, patients (or proxy) and caregiver will be asked to complete a questionnaire relating to their satisfaction with the treatment approach. In case a patient has died during follow-up, his/her relatives and caregiver will be asked to complete this questionnaire. In addition, relatives will be invited for an interview to complete the QODD questionnaire. An early withdrawal form will be completed if patients (or proxy) decide to withdraw from the study.

### Blinding

As with many surgical studies, patients and surgeons cannot be blinded for the intervention. In order to reduce bias as much as possible, outcome assessment will be performed using a standardized protocol. Outcome measures include both objective and subjective items.

### Statistical analysis

Data will be analyzed using the Statistical Package for the Social Sciences (SPSS) version 24.0 or higher (SPSS, Chicago, Ill., USA) and will be reported following the STrengthening the Reporting of OBservational studies in Epidemiology (STROBE) guidelines. Normality of continuous data will be tested with the Shapiro-Wilk test. Homogeneity of variances will be tested using the Levene’s test. A two-sided *p*-value < 0.05 will be taken as threshold of statistical significance in all statistical tests. The analysis will be performed on a per-protocol basis, (as that is the most conservative approach) and verified by an intention-to-treat analysis if needed. Patients who have died will remain in the analysis as censored. If necessary, missing values will be replaced using multiple imputation following the predictive mean matching method, using ten imputations.

Descriptive analysis will be performed in order to report the outcome measures and other variables collected for the entire population as well as per treatment group. Continuous data will be reported as mean and SD (if Normal) or median and quartiles (if non-Normal), categorical data as number with percentage.

Univariate comparison between the groups will be done using Student’s T or Mann-Whitney U-test (for Normal and non-Normal data, respectively), or Chi-squared test or Fisher’s Exact test (categorical data, as applicable). The Student’s T-test will be done with equal variance assumed or not assumed, based upon the results of the Levene’s test.

For the primary outcome measure, a non-inferiority margin of 0.15 points for mean EQ-5D scores was chosen because it is reasonable and on the border of what would be considered a clinically important effect. The null hypothesis in the non-inferiority framework is that nonoperative management is inferior to operative management with regards to the EQ-5D score. This hypothesis will be tested through the adjusted contrast between the two interventions at 6 months. Specifically, the 95% confidence interval of the estimated contrast will be examined, and if the limit of the interval is less than the threshold value of 0.15 points, non-inferiority of nonoperative management to operative management at 6 months will be concluded.

Multivariable analysis for continuous outcome measures that are repeatedly measured over time will be done using generalized estimating equations (GEE) for longitudinal analysis on a per-protocol and intention-to-treat basis to investigate the effect of treatment. In the primary GEE model, the primary outcome variable studied (quality of life on EQ-5D) will be analyzed as a dependent variable, using treatment (nonoperative versus operative) as between subjects variable and time as within subjects variable. The interaction term of group and time (group x time) will be assessed, to evaluate whether the change over time differed between groups. Analyses will be corrected for baseline differences and all models will be corrected for center of inclusion. Repeatedly measured continuous secondary outcome variables will be analyzed using similar GEE models. Continuous outcome measures only ones and dichotomous secondary outcome measures will be analyzed using multivariable linear regression models and logistic regression models, respectively.

Subgroup analysis are planned for patients with an intracapsular or extracapsular fracture.

The economic evaluations will be done from a societal perspective. Intervention costs and health care utilization costs until 6 months after inclusion will be measured in accordance with economic guidelines [45]. Cost prices of the standardized referral strategy will be determined by bottom-up micro-costing method. The economic evaluation will be performed in accordance with the intention-to-treat principle. Missing data will be imputed using multiple imputation by changed equations [46]. The Incremental Cost-Effectiveness Ratio (ICER) of nonoperative versus operative management will be calculated by dividing the difference in costs by difference in effects. A cost-utility analysis, with QALY (based on the EQ-5D utility score) as outcome measure, will be done as primary economic analysis. As secondary analysis, a cost-effectiveness analysis, with QUALIDEM as outcome measure, will be done. In order to account for the possible clustering of data, analyses will be performed using linear multilevel analyses [47]. Accounting for the possible clustering of data (e.g., at the hospital level) is very important, as most economic evaluations fail to do so, whereas ignoring the possible clustering of data might lead to inaccurate levels of uncertainty and inaccurate point estimates [47]. Bootstrapping techniques will be used in order to estimate the uncertainty surrounding the cost-effectiveness estimates. Uncertainty will be shown in cost-effectiveness planes and cost-effectiveness acceptability curves, and sensitivity analyses will be performed in order to test the robustness of the study results [48–50].

### Sample size calculation

The sample size calculation is based on a non-inferiority design, i.e., on the hypothesis that nonoperative management is non-inferior to operative management. As stated before the EQ-5D will serve as primary outcome measure. We have used the results from earlier studies on proximal femoral fracture treatment in the elderly for the sample size calculation [51–53]. Assuming a two-sided significance level (alpha) of 0.05 and a power of 80% with a standard deviation (s) of 0.30 on the EQ-5D utility score and a noninferiority limit (d) of 0.15 (which equals 0.5 SD), a total of 50 subjects are needed in each treatment arm. Taking into account a rate of 60% loss to follow-up and mortality until 3 months, a total number of 160 participants has to be recruited.

### Data management and monitoring

Data will be encoded and stored in a pass-word protected database with restricted access to the researchers only. Data will be entered once. Quality of the entered data will be monitored by checking entry for a random sample of patients prior to database locking.

## Discussion

Treatment guidelines for proximal femoral fractures are mainly focused on regaining function and independence in activities of daily living. Other considerations may apply to frail elderly with a limited life expectancy, for whom comfort and pain are particularly relevant. Whether or not operative treatment is needed in this respect is undecided. This study aims to investigate the value of nonoperative management versus operative management of proximal femoral fractures in a selected group of frail institutionalized elderly with a limited life expectancy.

There are some limitations to this study. First, selection bias could be a factor in this study, since patient or proxy opting for nonoperative management may arguably be in worse condition with regards to their pre-trauma condition and comorbidities. A randomized controlled trial (RCT) would have avoided selection bias, however randomizing patients with a limited life expectancy was considered unfeasible (and unethical) by the research team. Patient recruitment in trials randomizing between operative and nonoperative treatment is known to be challenging, and would likely introduce an unacceptably high level of selection bias; surgeons with clear preference for a particular treatment may be unwilling to inform patients about the study, and patients (or their proxy if patient is unable to decide) with preference for a particular treatment will not sign informed consent. The chosen observational design allows all centers and all patients to participate, and real-life data will be collected. For patients with severe cognitive impairment, informed consent will be obtained by their health care proxy. That makes the current study design the most feasible to investigate this issue. As a second limitation, it could be argued that the questionnaire completed by proxy may differ from the patients rating, however since the majority of this study population is expected to suffer from cognitive impairment, questionnaire completion by proxy will be inevitable. Finally, at least 26 hospitals will participate, and treatment protocol and shared decision processes may vary across sites. This may introduce some heterogeneity, but also makes the results more generalizable.

To the best of our knowledge, this is the first comparative study that addresses this issue with regard to quality of life and cost-effectiveness and also the first study to provide detailed information about nonoperative management with a longer term follow-up in frail patients with a proximal femoral fracture. This study will contribute widely to the current knowledge about the process of nonoperative management, treatment satisfaction with patients, proxy and caregivers in this select group of frail hip fracture patients in the context of quality of life. The results of this study may be used for updating the Dutch national “Guideline for the treatment of proximal femoral fractures” (set out by the Dutch Association of Surgery and the Dutch Orthopaedic Association) and the Dutch national “Guideline for multidisciplinary treatment of vulnerable elderly treated operatively” (set out by the Dutch Association of Clinical Geriatrics), but may also help updating international treatment guidelines on proximal femoral fractures. The results could be further used in the context of advanced care planning and expectation management and therefore aid treatment decision making in future frail hip fracture patients.

## Data Availability

Not applicable.

## Abbreviations

ADL: Activities of daily living
ASA: American Society of Anesthesiologists
BMI: Body Mass Index
CCI: Charlson Comorbidity Index
CT: Computated Tomography
ED: Emergency Department
EQ-5D: EuroQoL-5D
FAC: Functional Ambulation Category
GCP: Good Clinical Practice
GEE: Generalized estimating equations
ICER: Incremental Cost-Effectiveness Ratio
iMCQ: Medical Consumption Questionnaire
iMTA: Institute of Medical Technology Assessments
MTREC: Medical Research Ethics Committee
NICE: National Institute for Health and Care Excellence
NOV: Dutch Orthopaedic Association
NVKG: Dutch Association of Clinical Geriatrics
NVOT: Dutch Association of Orthopaedic Surgery
NVT: Dutch Association of Trauma Surgery
NVvH: Dutch Association of Surgery
PACSLAC: Pain Assessment Checklist for Seniors with Limited Ability to Communicate
PACSLAC-D: Pain Assessment Checklist for Seniors with Limited Ability to Communicate - Dutch language version
PFN: Dutch Patient Federation
QALY: Quality Adjusted Life Years
QODD: Quality of Dying and Death
QoL: Quality of Life
SD: Standard Deviation
SDM: Shared Decision Making
SPSS: Statistical Package for the Social Sciences
STROBE: STrengthening the Reporting of OBservational studies in Epidemiology
VERENSO: Association of Elderly Care Physicians
WHO: World Health Organization
WMO: Medical Research Involving Human Subjects Act (in Dutch: Wet Medisch-wetenschappelijk Onderzoek met Mensen
